# High Risk of ART Non-Adherence and Delay of ART Initiation among HIV Positive Double Orphans in Kigali, Rwanda

**DOI:** 10.1371/journal.pone.0041998

**Published:** 2012-07-30

**Authors:** Kimiyo Kikuchi, Krishna C. Poudel, John Muganda, Adolphe Majyambere, Keiko Otsuka, Tomoko Sato, Vincent Mutabazi, Simon Pierre Nyonsenga, Ribakare Muhayimpundu, Masamine Jimba, Junko Yasuoka

**Affiliations:** 1 Department of Community and Global Health, Graduate School of Medicine, The University of Tokyo, Tokyo, Japan; 2 Division of Obstetrics and Gynecology, King Faisal Hospital, Kigali, Rwanda; 3 HIV/AIDS, STIs and Other Blood Borne Infections Division, Institute of HIV Disease Prevention and Control, Rwanda Biomedical Center, Kigali, Rwanda; 4 Department of Psychology, The Meiji Gakuin University, Tokyo, Japan; Chancellor College, University of Malawi, Malawi

## Abstract

**Background:**

To reduce HIV/AIDS related mortality of children, adherence to antiretroviral treatment (ART) is critical in the treatment of HIV positive children. However, little is known about the association between ART adherence and different orphan status. The aims of this study were to assess the ART adherence and identify whether different orphan status was associated with the child’s adherence.

**Methods:**

A total of 717 HIV positive children and the same number of caregivers participated in this cross-sectional study. Children’s adherence rate was measured using a pill count method and those who took 85% or more of the prescribed doses were defined as adherent. To collect data about adherence related factors, we also interviewed caregivers using a structured questionnaire.

**Results:**

Of all children (N = 717), participants from each orphan category (double orphan, maternal orphan, paternal orphan, non-orphan) were 346, 89, 169, and 113, respectively. ART non-adherence rate of each orphan category was 59.3%, 44.9%, 46.7%, and 49.7%, respectively. The multivariate analysis indicated that maternal orphans (AOR 0.31, 95% CI 0.12–0.80), paternal orphans (AOR 0.35, 95% CI 0.14–0.89), and non-orphans (AOR 0.45, 95% CI 0.21–0.99) were less likely to be non-adherent compared to double orphans. Double orphans who had a sibling as a caregiver were more likely to be non-adherent. The first mean CD4 count prior to initiating treatment was 520, 601, 599, and 844 (cells/ml), respectively (*p*<0.001). Their mean age at sero-status detection was 5.9, 5.3, 4.8, and 3.9 (year old), respectively (*p*<0.001).

**Conclusions:**

Double orphans were at highest risk of ART non-adherence and especially those who had a sibling as a caregiver had high risk. They were also in danger of initiating ART at an older age and at a later stage of HIV/AIDS compared with other orphan categories. Double orphans need more attention to the promote child’s adherence to ART.

## Introduction

Worldwide, the AIDS epidemic puts children at grave risk. The Millennium Development Goals (MDGs) call for stopping the spread of HIV/AIDS by 2015, but progress has not been as fast as expected and approximately 370,000 children under 15 years old were newly infected with HIV in the world in 2009 [1]. In particular, HIV infection has severely hit Sub-Saharan African countries, where the number of HIV positive children represents 90% of all HIV infected children in the world [1]. In this region, HIV/AIDS has been the main cause of the highest under-five mortality rate [2], [3].

HIV infection among orphaned children constitutes an important issue in Africa. According to previous studies, orphans aged under 15 who have lost one or both parents constitute an especially vulnerable group of HIV positive children. Compared with non-orphans, orphans suffer from poorer health and nutritional status [4]–[7]. Furthermore, orphans initiate their antiretroviral treatment (ART) later than non-orphaned children, and they are more likely to be diagnosed as WHO Clinical Stage 4 (most severe HIV infection stage) prior to ART initiation than non-orphans [8].

To reduce children’s HIV/AIDS related mortality and progression, early ART initiation and high levels of ART adherence are particularly critical [9]–[12]. However, promoting high ART adherence in children is difficult due to various factors which derive from children’s developmental immaturity, their particularity of eating habits, and a lack of knowledge about their disease. Moreover, the adherence of children, especially younger children, is largely dependent on their caregivers, which makes children’s adherence matters more complicated than in the case of HIV positive adults. Well-known barriers identified in quantitative studies in Sub-Saharan Africa include: caregivers' perceived difficulty for administrating ART, child does not know his/her sero-status, and older age of child [13]–[15]. On the other hand, the relation between adherence and different orphan status (non-orphan, maternal orphan, paternal orphan, and double orphan) needs to be examined more. In most adherence related studies, orphan status was classified as ‘orphan’ or ‘non-orphan’ [16], [17]. This binary categorization may be valid in study setting where single orphans and double orphans were favored with similar social and economic conditions. However, in most resource-limited settings, the death of two parents had a much greater additive effect on the caregiving situation [9]. In addition, caregivers’ support to a child may be different between maternal orphans and paternal orphans due to the difference in each parent’s relationship with the child following their social and cultural setting.

HIV positive orphans are key priorities in Rwanda’s national HIV/AIDS response. In 2009, the number of children aged 0–14 living with HIV was estimated at 22,000 [11,000–34,000], which accounted for 12% of Rwanda’s total HIV positive population [18]. The estimated number of children aged 0–17 orphaned due to HIV/AIDS was 130,000 [98,000–180,000] in 2009 [18], and a large number of these orphans were considered to be infected through a mother-to-child transmission (MTCT). Among all HIV positive children who needed ART in Rwanda, 6,558 had initiated ART by the end of 2009 [19]. This represents 60% of the pediatric coverage, which ranks one of the highest in African countries [1]. However, no official study has been conducted in Rwanda about ART adherence which focuses on HIV positive children.

The objectives of this study were to assess the ART adherence among HIV positive children in Kigali and to identify whether different orphan status was associated with the child’s ART adherence.

## Methods

### Operational Definition

We defined caregiver as a person whom the child turned to when he/she needed to take medication. Caregivers of the child were identified by self-assessment before the interview. Caregivers may include the child’s parents, siblings, grandparents, other relatives, or guardians who directly influenced the child’s medication dosing.

As for orphan status, a double orphan was defined as a child who had no living parents or had no parents whose living status was known. A maternal orphan/paternal orphan was defined as a child whose mother/father had died or they didn’t know their living status. The cutoff point of the child’s age was set at 15 following the definition of UNICEF [20], [21] and that of pediatric patients in Rwanda.

### Study Design and Site

We carried out a facility-based, cross-sectional study between March and July 2011 at selected health facilities in Kigali, Rwanda. We counted remaining pills to assess the adherence rate of children currently enrolled in ART programs. To collect data about associated factors to adherence, we also conducted a questionnaire survey for primary caregivers, measured children’s weight/height, and collected clinical records of children. We choose Kigali city as the study site since the city enrolls a high number of pediatric ART patients (n = 1558) and runs a high percentage (23.8%) of Rwanda’s pediatric ART services [19]. To recruit participants from various facility levels, we used two sampling methods. First, all four public hospitals and one clinic in Kigali were selected since they enroll almost half of all pediatric patients in Kigali (TRAC-Plus Clinic, Kigali University Hospital Center, Muhima District Hospital, Kigabagaba District Hospital, and Kanombe Military Hospital). Second, out of 21 health centers which provide a routine pediatric ART service, we randomly selected 10. They were: Kimironko, Kinyinya, Kicukiro, Kabusunzu, Cor-unum, Kacyiru, Gitega, Gikondo, Masaka, and Biryogo.

### Study Population

The study population was children enrolled in the ART program in the 15 selected health facilities and their primary caregivers. Inclusion criteria for children in the study were: having been under ART for at least 12 weeks, and were aged six months to 14 years. The exclusion criteria were: under only cotrimoxazole prophylaxis. The caregivers were those who were caring for children who matched the above criteria and were over 18 years old.

### Measurements

#### Child’s adherence to ART

To measure ART adherence, we counted the remaining pills or syrups which participants presented on the day of the interview [22], [23]. Pill count measurement was used because of its objective and quantifiable advantage [24]. From client files, we collected data about the prescribed regimen, the last prescribed date, the pill quantity prescribed per day, and the number of days prescribed. Adherence was calculated based on the formula: [(pills dispensed/pills prescribed per day)/days between refills]×100. This equation has been used in prior studies to evaluate adherence to ART medication [25]. A child was defined to be adherent if he/she took 85% or more of the monthly prescribed doses. Those who were under 85% were defined non-adherent. This rate was estimated as a poor adherence according to the WHO guideline for adherence [16], [26], and more than a 85% adherence rate was reported as the minimum level to achieve HIV virus suppression and have clinical benefit [27]. For those who were taking multiple medications, a child was defined adherent if his/her adherence rate was at least 85% for each medication.

#### Questionnaires

We collected data from caregivers using a structured pre-tested questionnaire which was adopted from different studies [28]–[30]. The contents of the questionnaire included: socio-demographic characteristics of children and their primary caregivers, economic status of caregivers (income per month, number of rooms, water source, toilet facility, and electricity equipment), caregiver-child relationship, and caregivers’ knowledge about ART. To enhance the accuracy of translation, the questionnaire was originally developed in English, then translated into Kinyarwanda, and translated back into English. The questionnaire items were reviewed by three Rwandan experts on HIV/AIDS who were based in different health formations in Kigali. Prior to the survey, we provided two-day training for investigators to help them understand each question. The questionnaire was pre-tested for 20 caregivers and revised accordingly.

**Table 1:** Child Participant Characteristics Overall and Stratified by Orphan Status (N = 717).

Characteristics	Overall	Orphan Status				*p*-value
		Double Orphan	MaternalSingle-Orphan	PaternalSingle-Orphan	Non-Orphan	
N = (%)	717 (100)	113 (15.8)	89 (12.4)	169 (23.6)	346 (48.3)	
Health Facility						0.212
Hospital/Clinic	349 (48.7)	59 (52.2)	39 (43.8)	73 (43.2)	178 (51.4)	
Health Center	368 (51.3)	54 (47.8)	50 (56.2)	96 (56.8)	168 (48.6)	
Male	363 (50.7)	55 (48.7)	47 (52.8)	74 (43.8)	187 (54.0)	0.144
Female	354 (49.3)	58 (51.3)	42 (47.2)	95 (56.2)	159 (46.0)	
Age (yrs)						
<10	303 (42.3)	24 (21.2)	23 (25.8)	54 (32.0)	202 (58.4)	<0.001
≥10	414 (57.7)	89 (78.8)	66 (74.2)	115 (68.0)	144 (41.6)	
Duration on ART (yrs)						
<4 yrs	402 (56.9)	50 (46.3)	50 (56.8)	90 (53.3)	212 (62.0)	0.024
≥4 yrs	305 (43.1)	58 (53.7)	38 (43.2)	79 (46.7)	130 (38.0)	
Knows Sero-status among all children	361 (50.3)	75 (66.4)	57 (64.0)	94 (55.6)	135 (39.0)	<0.001
Disclosed Sero-status to Others	297 (41.4)	55 (48.7)	33 (37.1)	79 (46.7)	130 (37.6)	0.056

Caregiver-child relationship was measured using two of the eight scales from the Parent-Child Relationship Inventory (PCRI) [29]: communication and involvement. The PCRI was originally developed to evaluate the relationship between a parent and a child. However, it has also been used for measuring the caregiver-child relationship in a previous study which certified its validity and reliability [28]. Each score was converted to T-scores, and T-scores less than 40 were classified as low communication and involvement according to the PCRI guideline. Caregiver’s knowledge was assessed using a true/false self-report measure [30]. Caregiver’s knowledge level was estimated by the number of items which were correctly answered.

**Table 2:** Caregiver Participant Characteristics Overall and Stratified by Orphan Status (N = 717).

Characteristics	Overall	Orphan Status				*p*-value
		Double Orphan	MaternalSingle-Orphan	PaternalSingle-Orphan	Non-Orphan	
N = (%)	717 (100)	113 (15.8)	89 (12.4)	169 (23.6)	346 (48.3)	
Male	103 (14.4)	18 (15.9)	35 (39.3)	4 (2.3)	46 (13.3)	0.005
Female	614 (85.6)	95 (84.1)	54 (60.7)	165 (97.7)	300 (86.7)	
HIV Positive	531 (74.1)	18 (15.9)	42 (47.2)	157 (92.9)	314 (90.8)	<0.001
Age (yrs)						<0.001
<40	374 (53.0)	49 (43.8)	31 (35.6)	79 (47.0)	215 (63.2)	
≥40	333 (47.0)	63 (56.2)	56 (64.4)	89 (53.0)	125 (36.8)	
Duration on Caring Child (yrs)						0.001
<8	332 (46.7)	61 (54.5)	42 (47.2)	56 (33.1)	173 (50.7)	
≥8	379 (53.3)	51 (45.5)	47 (52.8)	113 (66.9)	168 (49.3)	
Relation with Child						<0.001
Parent	504 (70.3)	0 (0)	29 (32.6)	154 (91.1)	321 (92.8)	
Sibling	43 (6.0)	16 (14.2)	18 (20.2)	2 (1.2)	7 (2.0)	
Grand Parent	36 (5.0)	19 (16.8)	13 (14.6)	2 (1.2)	2 (0.6)	
Uncle/Aunt	92 (12.8)	51 (45.1)	23 (25.8)	8 (4.7)	10 (2.9)	
Orphanage Staff	16 (2.2)	15 (13.3)	1 (1.1)	0 (0)	0 (0)	
Foster Parents	8 (1.1)	4 (3.5)	1 (1.1)	2 (1.2)	1 (0.3)	
Other*	18 (2.5)	8 (7.1)	4 (4.5)	1 (0.6)	5 (1.4)	
Have Other Caregivers	314 (43.8)	53 (46.9)	60 (67.4)	26 (15.4)	176 (50.9)	<0.001
Have Formal Education	628 (87.6)	89 (78.8)	80 (89.9)	141 (83.4)	318 (91.9)	<0.001
Income per month (RWF)						0.540
0	352 (49.1)	55 (50.0)	49 (55.1)	74 (44.0)	174 (50.6)	
<26000	150 (20.9)	18 (15.9)	5 (5.6)	56 (33.1)	71 (20.5)	
≥26000	215 (30.0)	40 (34.1)	35 (39.3)	39 (22.9)	101 (28.9)	
Number of Rooms (Mean)	2.5	3.1	2.9	2.2	2.4	<0.001
Water Source						<0.001
Tap in House	247 (34.9)	61 (54.5)	36 (40.4)	40 (24.0)	110 (32.4)	
Shared Standpipe	346 (48.7)	37 (33.0)	42 (47.2)	90 (53.9)	177 (51.8)	
River	116 (16.4)	14 (12.5)	11 (12.4)	37 (22.1)	54 (15.9)	
Toilet Facility						0.002
Flush Toilet	33 (4.9)	13 (12.7)	4 (4.7)	2 (1.2)	14 (4.3)	
Pit Latrine	643 (94.6)	89 (87.3)	81 (95.3)	163 (97.6)	310 (95.1)	
No Toilet Facilities	4 (0.5)	0 (0)	0 (0)	2 (1.2)	2 (0.6)	
Have Electricity	445(62.1)	83 (73.5)	59 (66.3)	82 (48.5)	221(63.9)	<0.001
Parent Child Relationship Inventory						
SD of Involvement Score (Mean)	55.7	52.8	55.8	55.7	56.6	0.002
Low	66 (9.4)	21 (18.9)	8 (9.2)	12 (7.3)	25 (7.4)	
Normative	636 (90.6)	90 (81.1)	79 (90.8)	153 (92.7)	314 (92.6)	
SD of Communication Score (Mean)	62.4	64.3	63.5	64.2	60.6	<0.001
Low	38 (6.5)	0 (0)	2 (2.3)	5 (3.1)	31 (10.3)	
Normative	659 (93.5)	111 (100)	85 (97.7)	158 (96.9)	304 (89.7)	
Correct Answer for ART Knowledge						0.441
<6 score	236 (33.0)	44 (38.9)	31 (34.8)	54 (32.0)	107 (31.0)	
≥6 score	481 (67.0)	69 (61.1)	58 (65.2)	115 (68.0)	239 (69.0)	

* niece, cousin, neighbors, friend of parents, guardian.

#### Disease and growth status related data

We collected children’s latest medical records from client files, such as their CD4 counts, WHO Clinical Stage, as well as the date of the first HIV test and ART initiation. We also measured weight (kg) and height/length (cm) in all children. To measure them, we used portable electronic scales and metal measuring rods which were calibrated to 0.1 kg, 0.1 cm, respectively. *Z*-score of weight-for-age (WAZ), height-for-age (HAZ), and BMI-for-age were calculated using WHO AnthroPlus version 1.0.4 (WHO, Geneva. 2007). Undernutrition and stunting were determined using cut-off points of <−2 *Z*-score. To calculate WAZ and undernutrition, only children under 10 years old were included due to the data availability of AnthroPlus.

**Table 3:** Disease and Growth Status Related Factors Stratified by Orphan Status (N = 717).

	Overall	Orphan Status				*p*-value
		Double Orphan	Maternal Single-Orphan	Paternal Single-Orphan	Non-Orphan	
Adherence to ART						
Adherent (N (%))	362 (50.5)	46 (40.7)	49 (55.1)	93 (53.3)	174 (50.3)	0.039
Non-adherent (N (%))	355 (49.5)	67 (59.3)	40 (44.9)	76 (46.7)	172 (49.7)	
Mean CD4 count at first exam (cells/mL)	706	520	601	599	844	<0.001
Mean age at first HIV test (yrs)	4.6	5.9	5.3	4.8	3.9	<0.001
Mean age at ART initiation (yrs)	6.2	8.0	7.1	6.6	5.2	<0.001
WHO stage 4 at first visit (N (%))	55 (7.7)	11 (9.7)	8 (9.0)	12 (7.1)	24 (7.0)	0.486
Change of ART regimen (N (%))	173 (24.1)	28 (24.8)	24 (27.0)	31 (18.3)	90 (26.1)	0.225
Detection of child’s sero-status						
By PMTCT	387 (54.0)	42 (37.2)	42 (47.2)	96 (56.8)	207 (60.0)	<0.001
By child’s illness	340 (47.4)	69 (61.1)	46 (51.7)	78 (46.2)	147 (42.5)	0.006
						
Mean (SD) BMI-for-age	−0.43 (1.45)	−0.67 (1.44)	−0.43 (1.23)	−0.48 (1.35)	−0.33 (1.54)	0.191
Mean (SD) WAZ (<10 yrs, n = 292)	−1.05 (1.16)	−0.88 (1.15)	−1.10 (0.97)	−1.22 (1.08)	−1.01 (1.20)	0.596
Mean (SD) HAZ	−1.86 (1.47)	−1.73 (1.37)	−1.78 (1.34)	−2.08 (1.46)	−1.82 (1.53)	0.171
Undernutrition (<10 yrs, n = 292) (N (%))	61 (20.9)	3 (12.5)	6 (27.3)	10 (19.6)	42 (21.5)	0.645
Stunting (N (%))	316 (46.0)	42 (38.2)	37 (43.5)	85 (52.1)	152 (46.2)	0.145

### Data Analysis and Processing

For data analysis, we carried out descriptive analysis for each orphan status to explore all variables of the children and the caregivers. We used the chi-square test for nominal scale variables. ANOVA was used for interval variables. To examine associations between the exposure variables and non-adherence, we conducted a multivariate logistic regression analysis. Variables which showed *p*-value <.25 in the bivariate analysis were included the model [31]. We also entered additional variables which were associated with ART adherence in previous studies such as gender and age of child and caregiver, relation of caregiver to the child, caregiver’s sero-status, child’s duration on ART, and child’s awareness of sero-status. Statistical significance was set at p<.05. Data entry and analysis were executed using PASW Statistics version 18.0 (SPSS Inc., Chicago, IL).

**Table 4:** Factors related to ART Non-Adherence among All Participants (N = 717).

Characteristics	Adjusted Odds Ratio
Orphan Status	
Double Orphan	**1.00**
Maternal Single-Orphan	**0.31 (0.12–0.80)**
Paternal Single-Orphan	**0.35 (0.14–0.89)**
Non-Orphan	**0.45 (0.21–0.99)**
Gender of Child	
Female	1.00
Male	0.98 (0.69-–.40)
Age of Child (yrs)	
<10	1.00
≥10	0.77 (0.48–1.26)
Gender of caregiver	
Female	1.00
Male	1.13 (0.65–1.97)
Age of Caregiver (yrs)	
<40	1.00
≥40	1.00 (0.68–1.49)
Relation of Caregiver with Child	
Parent	1.00
Siblings	0.48 (0.20–1.14)
Grand Parent	0.69 (0.29–1.66)
Uncle/Aunt	0.73 (0.28–1.93)
Caregiver’s Sero-Status	
Positive	1.00
Negative	1.36 (0.68–2.74)
Pill Burden (total No. of pills per day)	
<3	**1.00**
≥3	**1.56 (1.09–2.23)**
Duration on ART	
<4 yrs	1.00
≥4 yrs	0.93 (0.65–1.33)
Involvement	
Normative	1.00
Low	**2.14 (1.13–4.06)**
Communication	
Normative	1.00
Low	1.47 (0.49–4.38)
Child Knows Sero-Status	
No	1.00
Yes	0.97 (0.62–1.52)
Knowledge about ART	
<6 score	1.00
≥6 score	1.41 (0.96–2.07)
Have Other Caregiver	
No	1.00
Yes	1.27 (0.86–1.89)
Have Disclosed to Others	
No	1.00
Yes	1.41 (0.98–2.03)
Stunting (HAZ<−2)(n = 687)	
≥−2	1.00
<−2	**1.46 (1.01–2.09)**
BMI for age	
≥−2	1.00
<−2	0.81 (0.45–1.44)

**Table 5:** Factors related to ART Non-Adherence among Double Orphans (N = 113).

Characteristics	Adjusted Odds Ratio
Gender of Child	
Female	1.00
Male	1.12(0.31–4.05)
Age of Child (yrs)	
<10	1.00
≥10	0.34 (0.06–1.82)
Gender of caregiver	
Female	1.00
Male	1.12 (0.20–6.20)
Age of Caregiver (yrs)	
<40	1.00
≥40	0.42 (0.10–1.70)
Relation of Caregiver with Child	
Siblings	1.00
Aunt/Uncle	**0.14 (0.03–0.84)**
Grand Parent	**0.09 (0.10–0.96)**
Caregiver’s Sero-Status	
Positive	1.00
Negative	1.47 (0.32–6.79)
Pill Burden (total No. of pills per day)	
<3	1.00
≥3	0.78 (0.25–2.47)
Duration on ART	
<4 yrs	1.00
≥4 yrs	0.93 (0.28–3.11)
Involvement	
Normative	1.00
Low	1.96 (0.50–7.68)
Child Knows Sero-Status	
No	1.00
Yes	1.34 (0.37–4.90)
Knowledge about ART	
<6 score	1.00
≥6 score	0.65 (0.20–2.15)
Have Other Caregiver	
No	1.00
Yes	0.95 (0.29–3.05)
Have Disclosed to Others	
No	1.00
Yes	0.69 (0.23–2.06)

### Ethical Consideration

We obtained ethical clearance from the National Ethics Committee of Rwanda and the Ethics Committee of the University of Tokyo. An official agreement of data sharing was issued from the Institute of HIV Disease Prevention and Control/Rwanda Biomedical Center to carry out the data collection in each facility.

We started interviews after obtaining participants’ written consent. As the children are non-emancipated, written consent was carried out at two levels. First, the caregivers gave written consent. Then, when the children assented to participation, the caregivers signed an informed consent in place of the children.

## Results

### Participant Characteristics

Out of 1,301 children under an ART program in the 15 study sites, 767 children and the same number of their caregivers were available during our interview period (Table 1). Among them, 50 participants were excluded from analysis due to uncountable remaining pills caused by mixing with other pills (n = 22), incomplete data (n = 17), and/or missing client file (n = 11). Finally, 717 were included in our study.

### Socio-demographic Characteristics of Children

Out of the total, double orphans, maternal orphans, paternal orphans and non-orphans were, 113 (15.8%), 89 (12.4%), 169 (23.6%), and 346 (48.3%), respectively (Table 1). Approximately half of the children were males (50.7%), and the mean age of children was 9.3 (SD 3.6) years old. Regarding duration under ART, the mean was 3.7 (SD 2.0) years. Those who knew their sero-positive status (*p*<0.001) were fewer among non-orphans than other orphan categories.

### Socio-demographic Characteristics of Caregivers

Of all caregivers, 85.6% were female and about three-fourths (74.1%) were HIV positive (Table 2). The mean age was 39.3 (SD 10.5) years old and the mean duration of child caregiving was 7.9 (SD 10.5) years. The relation of caregiver to the child differs significantly between the four orphan status (*p*<0.001). The majority of caregivers were the child’s parent among non-orphans and paternal orphans, whereas among maternal orphans’ and double orphans’ caregivers varied. Fewer paternal orphans’ caregivers had other caregivers who helped with child care (*p*<0.001). Regarding economic status, we found that double orphans were in a higher economic status and paternal orphans were in a lower economic status through the results of number of rooms in a household (*p*<0.001), water source (*p*<0.001), toilet facility (*p* = 0.002), and electricity equipment (*p*<0.001). Involvement of caregivers in child care was rather lower among double orphans (*p* = 0.002), while communication between the caregivers and the child was lower among non-orphans (*p*<0.001).

### ART Adherence and Disease/growth Status Related Characteristics of Children

Of all 717 children, almost half (50.5%) were taking 85% and more of their prescribed doses (Table 3). Those who didn’t attain 85% were categorized as the group of ART non-adherent. The non-adherent rate of double orphans, maternal orphans, paternal orphans and non-orphans were 59.3%, 44.9%, 46.7%, and 49.7%, respectively.

CD4 count at first examination (*p*<0.001) differed significantly between the four orphan status groups. Moreover, double orphans were more likely to be older at the first sero-status detection (*p*<0.001), as well as at ART initiation (*p*<0.001). According to the responses related to how children’s sero-status was detected, double orphans were more likely to be detected by the child’s frequent illness (*p* = 0.006), whereas non-orphans were more likely to be found by the PMTCT (prevention of mother-to-child transmission) program (*p*<0.001). Children’s growth status did not differ significantly across the orphan status.

### Factors Associated with ART Adherence

The multivariate analysis indicated that maternal orphans (AOR 0.31, 95% CI 0.12–0.80), paternal orphans (AOR 0.35, 95% CI 0.14–0.89), and non-orphans (AOR 0.45, 95% CI 0.21–0.99) were less likely to be non-adherent to ART compared to double orphans (Table 4). Children who were prescribed three or more daily pills (AOR 1.56, 95% CI 1.09–2.23) and those whose growth was stunted (AOR 1.46, 95% CI 1.01–2.09) were more likely to be ART non-adherent. In addition, children whose caregiver scored lower on the involvement scale were more likely to be ART non-adherent than those whose caregivers scored normative (AOR 2.14, 95% CI 1.13–4.06). Among double orphans, children whose caregivers were their aunt/uncle (AOR 0.14, 95% CI 0.03–0.84) and children whose caregivers were a grand-parent (AOR 0.09, 95% CI 0.10–0.96) were less likely to be non-adherent compared to children whose caregivers were a sibling (Table 5).

## Discussion

This study is one of the first to report pediatric ART adherence in Rwanda. This is also one of the few studies which found an association of ART adherence with four different orphan status. Findings reveal that more than half of the HIV positive children on treatment were adherent to ART. Double orphans were at highest risk of ART non-adherence and especially those who had a sibling as a caregiver had risk of non-adherence. They were also in danger of the latest initiation of ART due to the late detection of sero-status compared to other orphan status children. Furthermore, caregivers’ low involvement in child matters, taking more than three antiretroviral pills daily, and stunted children were positively associated with ART non-adherence.

ART adherence rate of our population was a rather moderate level compared with previous studies which ranged between 29% and 100% [32], [33]. In our study population, mean duration on ART was rather longer compared to previous studies which showed higher adherence rates [9], [16], [17]. The relatively long duration on ART among our study population might have affected low adherence to medication due to treatment fatigue. Our result demonstrated the need for further efforts to enhance ART adherence rate. For its practice, the following suggestions could be taken into consideration.

To the best of our knowledge, this is the first study to reveal that double orphans had higher risk of ART non-adherence compared to not only non-orphans, but also maternal orphans and paternal orphans. Although most of the previous studies compared “orphan” and “non-orphan” only, our result pointed out the importance of distinguishing between having both parents dead and having one parent dead, as well as between having a father dead and having a mother dead.

According to our study results, double orphans’ non-adherence risk was high when the caregiver was his/her sibling. As a previous study revealed, the sibling might not be capable of providing appropriate care for the child due to less matured skills and lower capacity for child care without adult’s supervision [34]. Another point why double orphans tended to be non-adherent could be related to the caregivers’ motivation. The non-biological caregiver of a double orphan may be less motivated to maintain caring for the child’s adherence and less affected by the guilt associated with MTCT compared with a biological caregiver. This was also endorsed by lower caregiver involvement in child care among double orphans’ caregivers.

Furthermore, our result demonstrated that single orphans had lower non-adherence risk compared to double orphans and non-orphans, and especially maternal orphans had the lowest risk of non-adherence among all orphan status in comparison with double orphans. This suggested that having only one surviving parent might affect them maintaining high ART adherence. Also, we could suggest that the living status of the father had an important effect on the child’s high adherence to treatment, which derived from a father’s decisive initiative in the family. Association between father’s high involvement in child care and better adherence was also reported in US study on pediatric chronic disease [35].

Our study identified the late initiation of HIV treatment among double orphans being due to late detection of sero-status. A Ugandan study has reported that compared to non-orphans, an orphan’s age was significantly older and their CD4 count was significantly lower at the initiation of ART [8]. However, different orphan status was not focused on in that study. As our study demonstrated, non-orphans tend to have their sero-status detected during the PMTCT program which monitors a child’s sero-status from their birth. On the contrary, double orphans were not detected till frequent illness emerged, which means they were at an advanced stage of the disease. This point is also endorsed by a significantly lower CD4 count among double orphans at the first examination. This result highlighted the vital need for early HIV detection specifically among double orphans.

Paternal orphans’ sero-status was detected earlier than maternal orphans. Also, their sero-status was detected more by PMTCT than maternal orphans. This suggested that the mother’s living status affected an earliness of the child’s sero-status detection. While a father’s existence influences a child’s better adherence, a mother’s existence might lead to earlier detection of a child’s symptom.

Children whose caregiver was strongly involved in the child’s matters were more likely to be ART adherent. This result is consistent with a study from the US which showed that better medication adherence can be achieved by greater caregiver-child relationships [10], [11]. Caregivers’ involvement may directly affect child care in monitoring ART adherence. Addressing the involvement of the caregiver may be a prominent component of interventions to improve adherence.

Pill burden (number of daily pills) was associated with adherence. The same result has been reported in previous studies [36]–[38]. Taking a larger number of medication and the complexity of administration impair appropriate adherence to the treatment. To promote adherence, we need to identify the difficulties perceived by children prescribed a large quantity of medication, and take necessary steps to address these difficulties. However, there are also other studies which found no association [17], [39], thus there still remains a need for more inquiry about the relation between pill burden and adherence.

The study revealed that stunting (HAZ<−2) was positively associated with ART non-adherence. This result suggested that non-adherence may restrain the child from gaining HAZ, since some studies showed the significance for improving HAZ after the initiation of ART [40], [41]. On the other hand, the association between stunting and non-adherence could also have resulted from a lack of food. Lack of food has been cited as one of the ART adherence barriers among pediatric populations in resource-limited settings [42]. Since Rwanda is one of the countries whose population suffers from insufficient nutritional status [43], lack of food could be an important barrier in non-adherence.

This study has several limitations. First, as the protocols for ART provision differs in each health facility, conclusions about individual adherence rates may be limited. In principle, a patient’s remaining pills were required to be collected by health staff on the day of a patients’ appointment and patients then get their monthly resupply and leave the clinic with the exact number of pills after each visit. However, this wasn’t strictly practiced. Second, as patients were sometimes provided with additional quantities of medicine to avoid unexpectedly missing doses, this may also have affected the resulting adherence rate. To minimize the influence of these two limitations, we asked participants their reasons for excess or deficiency of medicine as much as possible and examined the data.

### Conclusions

Our study revealed that double orphan status was at the highest risk of ART non-adherence compared to other orphan status, and especially those who had a sibling as a caregiver had the highest risk of non-adherence. Double orphans were also in danger of initiating their ART at an older age and at a later stage of HIV/AIDS due to delays in their HIV detection. We, thus, highlight the need for particular interventions for HIV positive double orphans by supervising their adherence intensively and detecting their HIV infection at an earlier stage. Moreover, we demonstrated that adherence was associated with various factors such as a poor caregiver-child relationship, pill burden and the child’s poor nutritional status. To improve ART adherence, it is crucial to implement interventions that can promote better involvement of caregivers in child care, support children prescribed a large quantity of pills, and improve child nutrition.
